# The Abortion Plot: A Lyrical Critique of the Prosecutions for Suspected ‘Illegal’ Abortion in England

**DOI:** 10.1007/s10691-025-09589-w

**Published:** 2025-12-17

**Authors:** Ella Berny

**Affiliations:** Queen Mary University of London, London, UK

**Keywords:** Abortion, Abortion law, Abortion prosecutions, Reproductive justice, Language, Plot

## Abstract

This poetic/critical piece offers an urgent reflection on nine criminal prosecutions for suspected ‘illegal’ abortion in England that occurred between 2012 and 2025. Drawing on published traces of the cases, including sentencing remarks, appeal decisions and media reports, this essay-meditation evokes the haunting, suffocation and cruelty imposed by the criminalisation of abortion. In paying attention to plot and the discursive violence of this particular legal framing, this piece applies creative writing methods to provoke a destabilising, denaturalising, twisting, deplotting, dismantling and undoing, in a project of reproductive justice, abolition and liberation. CONTENT WARNING: This piece contains distressing content taken directly from prosecutions and trials, including references to abortion, stillbirth, infant death, murder, domestic violence.

## Notes on the Text

On 17 June 2025, British MPs voted to partially decriminalise abortion access for women in England and Wales, via amendment NC1 that was adopted into the Crime and Policing Bill at report stage. Currently, abortion in England and Wales remains in the criminal code, governed by the Victorian-era Offences Against the Person Act (OAPA) 1861 which carries a sentence of life imprisonment. The Abortion Act 1967 introduced grounds via which abortion became legal up to 24 weeks, but did not scrap the 1861 legislation. The recent vote means that once the Crime and Policing Bill is passed, women will no longer be prosecuted or sent to jail for ending their pregnancies outside the parameters of the Abortion Act. It will not, however, repeal the OAPA or fully decriminalise abortion. It will not mean that everyone is exempt from criminal investigation and prosecution for abortion related ‘offences’. People who support and provide abortion – doctors, midwives, accompaniers, doulas, partners, family, friends – will not be exempt from punishment for assisting an abortion that occurs beyond the conditions of the Abortion Act. And it is likely that such prosecutions will continue to take place.

In recent years, there has been a rise in the number of prosecutions for suspected ‘illegal’ abortion in England. Six women have been prosecuted since December 2022, suspected of self-administering abortion beyond the legal time limit (Oppenheim and Patrick 2024). Previous to this, it is believed that only three women had been convicted of having an ‘illegal’ abortion since the introduction of the Abortion Act in 1967 (Oppenheim and Patrick 2024). There has simultaneously been an ‘unprecedented’ (Jonathan Lord quoted in Malone 2024) rise in criminal investigations into suspected ‘illegal’ abortion, coinciding with the legalisation of at-home administration of abortion medications (mifepristone followed by misoprostol) in 2020, which has potentially increased awareness and suspicion amongst police and medical professionals (Sheldon and Lord 2023, p. 524; Tongue 2024).

This poetic/critical piece offers an urgent reflection and meditation on these prosecutions, including the most recent trial of a woman acquitted in May 2025. It draws on published traces of the cases, including sentencing remarks, appeal decisions and media reports. All italicised passages are direct quotes reflecting the words of those being prosecuted, the prosecution, defence, and witnesses.

Together, the published traces of these cases provide an overwhelming sense of the suffocation and cruelty imposed by the abortion law and the carceral state. Together, these traces bring to the surface ways in which legal framings (re)produce a certain discursive plotting of abortion: ways in which abortion is (re)produced as acceptable or unacceptable/good or bad. I draw on the word ‘plot’ in its multiple evocations: storyline, narrative, cunning, deceit, burial. In paying attention to plot and the discursive violence of this particular criminalisation and legal framing, I write with the intention of destabilising, denaturalising, twisting, deplotting, dismantling and undoing in a project of reproductive justice, abolition and liberation.

The recent passing of NC1 was celebrated, by many, as a victory for women’s rights. This ‘win’, however, comes within the context of the Crime and Policing Bill; a concerning proposal which aims to intensify the criminalisation of poverty and immigration, extend police powers, and further impose restrictions on the right to protest. This will include, as we’ve already been witnessing, increased policing and suppression of protests against Israel’s ongoing genocide in Palestine. NC1 also specifies the exemption of ‘women’ from criminal prosecution for abortion, calling into question what this means for trans people and whether such protections extend to them. Applauding NC1 therefore means accepting and validating an increased state of criminalisation, surveillance and oppression. It means accepting that some bodies count for more than others. It means accepting an intensified suppression of opposition to genocide. NC1, in this context, is a fallacy. Greater freedom for some bodies shouldn’t come at the expense of the increased policing, restriction and incarceration of others. This is not freedom at all. This is no victory for reproductive justice (see Ross and Solinger 2017; Doulas Decolonising 2023; Olufemi 2020: 38–46).

Criminalisation will continue to haunt bodies, including those who have abortions, including those who help people have abortions. It will continue to organise reproduction, hierarchising which bodies can reproduce freely, and which can’t. It will continue to haunt our bodies, even when we don’t realise, even when its effects seem naturalised. In highlighting the violence of the criminal law here, I hope to contribute to literature and activism that seeks reproductive justice beyond the institutions and systems that continue to threaten access and equity, through the imagining and creation of ‘transgressive spaces’ in which ‘care thrives, multiverses exist, and feminist transformations are truly possible’ (Nandagiri and Berro Pizzarossa 2023, p. 7).

* The names of those prosecuted or facing criminal investigation are anonymised in this piece,* including in references*,* to help protect the privacy of those who have had their privacy denied by the law. Pseudonyms marked with asterisk are used throughout to draw attention to this violation.*

ABORTION PLOT *in the courtroom*

Reading the public transcripts of sentencing remarks, appeal decisions and media reports of those prosecuted for suspected ‘illegal’ abortion in the UK in recent years brings me back to a nightmare where my scream, below water, is silent, so no one comes to help. A wave has pushed me under and now the currents dictate my return, but my voice doesn’t work below the surface. The more I try to call out and make a sound, the more the water comes flooding in to choke. I imagine

being these women**, in the dock or being addressed over video link. The architecture of law here smells thick. I imagine being pushed under and held down until I learn to hold my breath or grow gills or

become the current. I want to reach down and find their hands and pull them up so they can scream again. I want to tell them that no, you didn’t jump in with culpability, you were pushed in and drowned. The guilt, and remorse and pain you feel are the sedatives that were prescribed. No, you didn’t jump in. There was no choice, but to let your lungs

fill.

A (RE)PLOTTING

In the published traces of court we barely hear her* voice. Their voices. In the sentencing remarks, the appeal decisions, her words are sometimes recalled by the judge, through snippets of police interviews, or through the internet search terms she once typed. A ventriloquist speaks here, whilst her muted story gets told, and retold, and retold to her, until she herself is saying she is *preparing … for a prison sentence* and *deserve[s]* it.^1^ The plot

is working nicely, thinks the ventriloquist, who appears to speak truth whilst plotting through law. For in these walls her story is narrated through the canon of legal scripture. Replotted through its genre of divine antiquity and linearity and punishment. Hers is a bad story, a story-gone-wrong, replotted and retold and replotted until she loses her own plot and so holds on to what she’s being told is the right story, the wrong story, the only story that counts if she wants to be

free? *I felt I had no other choice other than to [plead guilty]. The prosecution said if I didn’t plead guilty*,* they would charge me with child destruction*,* and I would likely go to prison for life*, she said, upon release.^2^ Plead guilty to taking abortion pills to cause an illegal abortion or be charged with child destruction: which plot will you

choose? This is a choice, after all, the ventriloquist reminds. Are you pleading guilty to administering poison with intent to procure a miscarriage contrary to Sect. 58 of the Offences Against the Person Act 1861, or shall I charge you with Child

Destruction? You choose, my dear. Which plot will you choose? The judge consults the law book for his script. You see, *the balance struck by the law between a woman’s reproductive rights and the rights of her unborn fetus is an emotive and often controversial issue. That is*,* however*,* a matter for Parliament and not for the courts*, he explains.^3^ So there is really nothing he can do other than consult the

rules which are written in scarlet ink. In these walls. Dagger ink strokes, which permit only a certain way of telling. A sticky telling that clings

beyond. Out There, she may have been desperate, alone, terrified, forced to abort a future that haunted her, but In Here she *destroyed her child*. In Here, her story is rescripted through the *archaic language of an old Act of Parliament*^4^. In Here, she *administered poison* to *destroy*

*a child*. And now she is Poisoner, Poisoned, Killer.

*Mother jailed for taking poison to kill unborn son*, the headlines (re)told.^5^ And now, Out There, she is Poisoner, Poisoned, Killer.

IN THE PUBLIC INTEREST

She’s told that her privacy is in the public interest. In the dock, over video link, she listens as her life, as details of her sexual relationships, her private messages and internet searches, her obstetric history, her medical records, her darkest moments, the moments she has tried so desperately hard to conceal, are repeated back to her as if it doesn’t matter, as if it’s matter-of-fact. She watches as the jury pours over intimate pictures of her naked body, as they scrutinise the size of her nipples, as if it’s matter-of-fact-of-law. Matter-of-fact-of-public-interest. It’s repeated back to her, to her face, to the room, the public, her family and friends and neighbours, to journalists, through newspapers and TV screens and the radio, to her children’s school teachers, her children’s friends, her children. Now they all know that she is

*aged 44* [with] *no previous convictions. This offence was committed against the backdrop of the first and most intense phase of lockdown at the start of the COVID-19 pandemic.* The judge looks up to face her. *Forced to stay at home*,* you moved back in with your longterm but estranged partner while carrying another man’s child. You were*,* I accept*,* in emotional turmoil as you sought to hide the pregnancy. You are wracked by guilt and have suffered depression. I also accept that you had a very deep emotional attachment to your unborn child and that you are plagued by nightmares and flashbacks to seeing your dead child’s face.*^6^ She is silent as she listens

to a story stolen from her, told to her, as the words *your dead child’s face* reverberate. Words stab here, used to find law’s truth, not to care. Language is stripped, and the judge is justified by the scarlet ink which dictates how this story gets told. Don’t question. Don’t veer from the inked script. So the ventriloquist stares at her blankly, whilst he recounts, to her, to everyone, her innermost trauma, whilst he conjures the image of her

*dead child’s face* and

forces her to Face

It. It was your *tragic and unlawful decision*, he jibes.^7^ It was your *cold calculated decision that you took for your own convenience and in your own self interest alone*.^8^ I want to hold her hand and say

no. These walls are responsible for this pain and guilt, not you. It’s these walls and the rules of their bricks that forced this trauma, not you. Not

you.

The trial had to adjourn multiple times as Nancy* became distressed as details of the birth were discussed. 9

*Knowing this case could be made public and the implications we could face compounds fear*,* anxiety*, stress and uncertainty for Rosie* and our family.^10^

Nancy* cried as the prosecution detailed her account to police of losing the baby in the bathroom of her family home…while relatives were away. 11

Lois* wept throughout the sentencing hearing. 12

LEADING ROLES

The legal plot is character heavy. The Fetus and The Mother have leading roles. The Fetus morphs from case to case, sometimes taking centre stage, sometimes appearing to hang at the edges, but always there, a weighted presence. On occasions, The Fetus is the victim-protagonist, painted as *vulnerable and defenceless* and *an apparently healthy child*,* rob[ed]…of the life which he was about to commence*.^13^ Here, She is framed as Killer: *had he been born safely within a matter of days*,* and had you killed him after birth*,* you would be facing a charge of murder*, he jeers.^14^ Elsewhere, The Fetus appears as *Life*

*About To Begin*. Which has been *extinguished*.^15^ Or as *her unborn foetus* or *unborn child*, or is referred to as Daisy*, the name given by a mother to her stillborn baby.^16^ Whatever the level of fetal personhood ascribed to the character, a sense of personhood is undeniable and hangs thick, as the image of *your dead child’s*

*face* looms in the air. And she, standing there in the dock, the other, becomes The Mother, The Immoral Mother, The Un-Mother, The Killer-Mother, against this fetal personification.

Helen*, *the critical element of your offending is the deliberate choice made by you in the full knowledge of the due date.*^17^

Helen*, *what you did was to end the life of a child that was presumptively capable of being born alive.*^18^

Lois*, your *baby* would have had *a very good chance of survival*,* but had no chance once you administered this drug.*^19^

Rosie*, *in my judgment*,* your culpability is high;*

Rosie*, *you deliberately lied in order to bring yourself within the telemedical service for early medical abortions;*

Rosie*, *you had considerable previous obstetric experience; and there was some planning.* 20

Helen*, *had he been born safely within a matter of days*,* and had you killed him after birth*,* you would be facing a charge of murder*.^21^

The papers called him baby boy Jake.^22^

A CRIME OF TIME AND CHOICE

The law plots abortion as a crime of Time and Choice. The characters are neoliberal subjects, ruled by the biomedical clock and responsible by individual decision. The prosecution is an investigation of weeks and days. How many weeks? What gestation? How many days? When did she know? How far along was she? When did she get the pills? When did she take them? How pregnant did she think she was? How pregnant was she? When did she get the pills? When did she take the pills? What did she tell the clinician? Did she lie? Can we trust her? When did she take the pills? How many weeks? How many

days? Rosie*, *your search on 25 February indicated that you then believed that you were 23 weeks pregnant. Your internet searches continued sporadically through March and April 2020. On 24 April*,* you searched “I need to have an abortion but I’m past*

*24 weeks”.*^23^ When did she know? How far along was she? When did she get the pills? When did she take them? How pregnant did she think she was? How pregnant was she? When did she get the pills? When did she take the pills? What did she tell the clinician? Did she lie? When did she take the pills? Can we trust her? How many weeks? How many

days? *The information she gave to BPAS led them to conclude she was about 7 weeks pregnant…[She] took the abortifacients BPAS provided to her by post*,* and gave birth at home …to [a baby]. [The baby] was*

*stillborn.*^24^*In a text conversation with a friend at about the same time*,* she said: “I’m too old and I don’t want to be a single*

*mum”.*^25^ Her body, her breath, temporally

dislocated. Nancy* was *scared*,* stunned*,* catatonic almost* when the clinic told her she was 4 weeks 5 days too late.^26^ Not efficient enough. Unproductive. Temporally dislocated yet forced to breathe to the beat of the courtroom’s clock, a tick tick of medicine and biology, she is forced to walk to its metronomic

beat. *I don’t want to be a single*

*mum.* The surge is strong now as their hands fall deeper and deeper below, as the bubbles of their voices pop and evaporate at the surface, as the room fills, keeps filling, so that when

Helen* tries to recall her history of *disturbance*,* personal misery and entrenched problems*, 27 when

Rosie* recounts the *emotional turmoil*,^28^ when

Hannah* explains the *horror* of realising she was *much further along in the pregnancy* than she *could have possibly known*, feeling *terrified*, with *no words to describe* what she went through,^29^ when

Laura* tries to explain about her abusive boyfriend, who looked at her like she was a *rabid dog*,* grabbed hold of [her]*,* pushed [her] against the wall [and banged her] head off the wall*, how she was forced and *almost died* when *the bath filled with at least an inch of blood*, how she *wanted to die*, feeling like t*he whole world had just*

*ended in front of [her] eyes*,^30^ they

open their mouths, but

choke, as their living rooms fill with police, as the midwife calls the authorities, as she gets taken from hospital to the station, phone seized, still bleeding, as

the water

fills their lungs, as they realise time got the better of them, their

temporal contortion forced to reshape into the metronomic suffocation of the clock’s tick tick tick. As they realise

the law doesn’t care. As they are told they made a *deliberate choice*, that they *deliberately lied*, that *what you did was to end the life of a child*, that

whilst you are haunted by what happened to you, you

have now become Un-Mother, Killer-Mother, and must face that you made

*a choice*. I try to

call down to tell her, no, this was not a choice. This was a suffocation. An impossible situation with impossible outcomes. A *tragic accident.*^31^ A *stillbirth*.^32^ You should have been able to ask for help. I want to hold her hand, but the water is too thick, and she’s too far

down. As she

hears the words that will keep her pressed

under:

Helen*,* This is so serious that only an immediate sentence of imprisonment will suffice. The sentence will be one of 8 years.*^33^

Rosie*, *For the offence of administering poison with intent to procure a miscarriage*,* I sentence you to 28 months’ imprisonment.*^34^

DEFENCE: THE REGRETFUL FOETAL MOTHER

The defence in the Appeal focuses on rebuilding her character, making her a mother-more-palatable, or woman vulnerable, subject to tragedy and in need of paternalistic care. This is often down to her role as The Mother. Can she be scripted to fit the legal plot? Can she play a Mother *in-the-public-interest*? Can her Bad Abortion be made eloquent as one Good-Enough?

Helen* has two young children to whom it is accepted she is a good mother and whose development will be adversely affected by her absence from the family home. 35

I’m pleading with the court to show leniency… Rosie* is an upstanding member of the community. Irreplaceable in everyday life. Generous and thoughtful. Kind and caring a brilliant mother to our three sons. 36

*The two youngest of her children are in their teens. One of her children is autistic and needs her at home.* 37

*If I had known I was that far along I wouldn’t have done it… I wouldn’t have put the baby or myself through it.* 38

For The Mother role to be fully met, to be believed, she must be rid of culpability and intent through remorse and regret and grief and shame. The Defence must show she didn’t take the pills, or didn’t know the timings, or was emotionally unstable, vulnerable, lost and alone, breaking down, depressed, unable to cope, fragile, emotionally

immature.^39^ And sorry, so sorry for what she has done, so full of remorse and regret and grief and shame at what she has done, so sorry, and not inhumane, not un-motherly, a sad sorry mother who will do better for her current or future children, but nonetheless a mother, she is a mother, please believe us, she is and will be a

mother. It’s

in the public interest,

afterall. And

the courtroom wraps around her in an avuncular scarlett hug as

the ventriloquist recounts Helen’s history of *disturbance*,* personal misery and entrenched problems* and how she *lacks maturity in relation to emotional demand* and has *a maladaptive coping style*;^40^ and

Lois’s* suffering of *depressive episodes* and tendency to be *emotionally unstable;*^41^ and

how Rosie* shows *symptoms consistent with emotionally unstable personality traits*.^42^ And

the courtroom sighs a sigh of relief as it feels the plot resolving, as she slots into her part nicely, validating the scripture through the ventriloquist’s smiling acknowledgement of her

*plea of guilty*;^43^ through his acknowledgement of how

Helen* appears v*ery remorseful*, and feels she has *damaged many people’s lives by her actions*;^44^ of how

Lois* is *remorseful* and *greatly regrets her actions* and will live with *remorse* for *the rest of her life*;^45^ of how

She looks back and thinks *it’s awful*,* I hate myself*,* I hate it and I know it’s my fault*;^46^ of how

Rosie* is *aware she made a bad decision* and *will live with regret*,* guilt and loss for the rest of her life*;^47^ how she

*is ready to accept the consequences for her decision*,^48^ and

is *preparing herself for a prison sentence*, believing she *deserve[s] to go to prison*.^49^ He

smiles at his work, his masterpiece. And

the courtroom’s wrap closes in more tightly as he whispers to her: don’t worry, my child, I will save others from your mistakes, the law

will save us from

your sins.

OUT OF SYNC, AN UNRAVELLING?

But something is happening here, some unravelling. Some unravel-

ing.

As investigations mount and the law jibes harder, the repetition at once solidifies the structure and exposes cracks in the walls. Or

were the cracks always there? A seepage, inbetween, a dissolve-

ing. Something is happening to that scarlett ink, it’s been hit by a shard of sunlight and the glint has caught an eye, some eyes, and

seeing is becoming different, differently, is become-

ing. That glint has found all the gaps and fissures and cracks and is filling them with pinky-red light and is pushing through, through to the other side, or is it the same

side? Did the glint begin with the ink, or with the sunlight? No, that’s not the point. There is no beginning, just become-

ing. Something is out-of-sync, a twist in the plot, perhaps, the scarlet fading, perhaps, though still holding the page, none-the-less.

*It is a case in our view that calls for compassion*,* not punishment*, and it is one where no useful purpose is achieved by detaining Rosie* in custody.^50^*After assessing harm and culpability…we considered [the sentence] should be suspended… Rehabilitation had already been achieved*. Rosie* *presented no risk to her family or the wider public*,* and there was no prospect of a repetition of this offence*.^51^

Chloe*, the charges against you are being dropped. The prosecution is *not in possession of key evidence* and there is *no real prospect of conviction.*^52^

Nancy*, *we have concluded that it is not in the public interest to pursue further charges.*^53^*It has been a long and painful journey. The baby was stillborn in a bathroom and a life was sadly lost. The loss of that life was a tragedy*.^54^

Recorder Hardy questioned if the CPS’s public interest test was met in the case. *The public interest was in the baby remaining with the mother*, he said, *which might be at risk if she was convicted and sent to prison… This case in my judgement beggars belief*, he said.^55^

Have the cracks and fissures exposed the pain and the lack-of-public-interest, perhaps? Is something scrubbing away at that

ink? *It’s time to decriminalise!*^56^, they cry. Disbelief and calls for compassion seep into the courtroom, it seems. But…

*Our duty is to apply the law set by parliament impartially*, a CPS spokesperson said, again.^57^ It’s beginning to sound like blame, or deflection. *This was a really sensitive and*

*difficult case*, said a spokesperson for Shropshire* police, *which has been upsetting for all involved. After concerns had been raised to police by a healthcare professional*,* we had a duty to establish what had happened and carry out a full and impartial investigation*.^58^*A duty*

to apply the law, a *duty* to *carry out a full and impartial investigation*, despite not being *in the public interest*, despite being *upsetting for all involved. A duty* to apply the law, *a duty* to investigate, *a duty* to upset all involved, *a duty* to *retraumatise*,* a duty* to force them *to relive* traumatising events, *a duty* to mark bodies with suspicion and illegality, *a duty* to define and hierarchise the bodies that matter, *a duty* to uphold the carceral state, *a duty* to see her become *completely*

*broken.*^59^*It is never our intention to publicly embarrass or humiliate anyone with the evidence we put forward*, the CPS said. But

you did. But

you

did. So

the scarlet ink holds

tight, will not be scrubbed. *The defendants pleaded guilty to two offences*,* and after careful reflection we have concluded that it is not in the public interest to pursue further charges.*^60^ But by now the

damage is done, or is

be-ing done,

BEYOND THE COURTROOM

-ing, a constant

violence within and beyond the court’s walls. Chloe was not in court to hear her acquittal. *She has suffered so extensively over this prosecution and investigation*, her barrister said, that she couldn’t face it. She had been interviewed by police i*n the throes of grief* and investigated for three years.^61^ The trauma can not be un-

done. The walls of the

court do not contain. The courtroom is not a container. There is no beyond. It is everywhere and everything. It is every time, every text message, every clinic, every street, every bed, every bathroom, every internet search, every log in a period-tracker app, every uterus, every pregnancy, every pregnancy end. And as the wave

surges with more intensity, more and more debris gets swept along as more and more lives become collateral

damage. Like Sammy*, who delivered a baby at 26 weeks, born

blue, at home; who attempted CPR whilst waiting for an ambulance, only to be met by police, as well as paramedics; who, whilst caring for her new baby at hospital was met by officers who took her phone, and was a few days later taken into the station for questioning; who was told she and her husband were being investigated under suspicion of carrying out an illegal abortion, because Sammy* had considered an abortion during her pregnancy and had put pills in an online basket, although had never pressed the button and purchased them. *From the get go*,* we were just tret like*

*criminals*, she said.^62^ Becoming collateral

damage, like Aisha*, who delivered a baby prematurely at home, having experienced similar complications in earlier pregnancies; who told hospital staff she had considered an abortion at an earlier stage of pregnancy and so found herself interviewed under police caution; who was cut off from family and friends as her phone and tablet were confiscated; who was denied unsupervised access to her baby in the neonatal intensive care unit and so had to hand over expressed milk to the receptionist.^63^*From the get go*,

*we were just tret like criminals*, she said. The collateral

damage be-

ing done, as the tide surges in and the waves reach higher and higher and thicker and fuller, and the debris gets whipped up and churned around, and more and more debris gets pulled up and thrust around and shaken inside out and discarded. And the fear of being

whipped up in the debris path becomes deeper and deeper until it feels safer to keep quiet, to not speak out, or seek help, for fear of the risk, for fear of being pulled into the surge, for fear of choking, for fear of drowning. *What on earth made you think that medical professionals wouldn’t help you?* The prosecution demanded.^64^ The damage is be-

ing done, a constant become-

*But they didn’t really help me*,* did they?* she said. *So I think*

*I was right to be frightened.*^65^

-ing, as the ripples of suspicion and hostility reach further and further, salting the air with shame and immorality and fear; reaching further and further from the surge and appearing more and more a natural effect of the tide. Appearing more and more a natural affect of the tide. Appearing more and

more natural. Appearing

natural.

Until,

ENDS.

**All of the cases studied here prosecute cis women for suspected ‘illegal’ abortion so I often use the terms women/she/her collectively, whilst acknowledging that pregnancy, abortion and criminalisation affects trans and nonbinary people also (and acutely). Part of the violence of criminalisation comes from the specific and narrow gendering of reproductive bodies which demands a critique, for it to be undone/imagined otherwise.

## Footnotes

1 Rosie*’s words, recalled by Dame Victoria Sharp in *R v Rosie**, Appeal Judgment, [2023] EWCA Crim 1196 (18 October 2023) at par 14.

2 Laura’s* words, from an interview in 2022 after release from serving a two-year prison sentence, see Al-Othman 2022.

3 Words of the Hon. Mr Justice Pepperall, *R v Rosie**, Sentencing Remarks, (Crown Court Stoke on Trent, 12 June 2023) at para 1.

4 Words of Lady Justice Rafferty, *R v Helen**, Appeal Decision, [2013] EWCA Crim 1187 (12 June 2013) at para 18.

5 Hartley-Parkinson 2015.

6 Words of Pepperall J, in *R v Rosie**, 2023, (*supra* n 3) at para 20.

7 Words of Pepperall J, in *R v Rosie**, 2023, (*supra* n 3) at para 1.

8 Words of the Hon. Mr Justice Cooke, *R v Helen**, Sentencing Remarks, (Crown Court Leeds, 17 September 2012) at para 7.

9 Davis 2024.

10 Words taken from a letter written by Rosie’s* former partner, read out in court as part of her appeal case, *R v Rosie**, 2023, (*supra* n 1) at para 34.

11 Morris 2024.

12 Hartley-Parkinson 2015.

13 Words of Cooke J, *R v Helen**, 2012, (*supra* n 8) at para 15.

14 Words of Cooke J, *R v Helen**, 2012, (*supra* n 8), at para 14.

15 Words of Mr Justice Jay in the case of Lois***, quoted in Hartley-Parkinson 2015.

16 Words of Pepperall J, *R v Rosie**, 2023, (*supra* n 3) at paras 1, 20, 5 respectfully.

17 Words of Cooke J, *R v Helen**, 2012, (*supra* n 8) at para 2.

18 Words of Cooke J, *R v Helen**, 2012, (*supra* n 8) at para 12.

19 Words of Mr Justice Jay the case of Lois***, quoted in Hartley-Parkinson 2015.

20 Words of Pepperall J, *R v Rosie**, 2023, (*supra* n 3) at para 16.

21 Words of Cooke J, *R v Helen**, 2012, (*supra* n 8) at para 14.

22 For example see Spillett, Richard. and Brooke, Chris. 2015.

23 Words of Pepperall J, *R v Rosie**, 2023, (*supra* n 3) at para 3.

24 Words of Dame Victoria Sharp, *R v Rosie** (Summary of the Decision of the Court of Appeal, Criminal Division, 18 July 2023) p.2. Available at https://www.judiciary.uk/wp-content/uploads/2023/07/R-v-Carla-Foster-190723.pdf [accessed 6.8.25].

25 Words of Sharp, *R v Rosie**, 2023 (*supra n* 1) at para 6.

26 A clinical care coordinator at the BPAS clinic Nancy* visited describes her as being ‘shellshocked’ and ‘scared, stunned, catatonic almost’ when she realised she was over the legal limit for having an abortion. Morris 2024.

27 Words of Rafferty J, *R v Helen**, 2013, (*supra* n 4) at para 3.

28 Words of Sharp, *R v Rosie**, 2023, (*supra* n 1) at para 46.

29 Hannah*’s words, from her Crowd Justice webpage fundraising for legal fees, ‘I face trial for having an NHS prescribed abortion. I need your help’. <https://www.crowdjustice.com/case/notacrime/%3E [accessed 19.5.25].

30 Laura’s* words, quoted in Al-Othman 2022.

31 Hannah’s* words, quoted in Al-Othman 2025. ‘‘I was right to be frightened’: Hannah* on the humiliation and trauma of her trial for illegal abortion’, *The Guardian.* 14 May. [Accessed 22.5.25].

32
*R v Rosie**, 2023 (*supra* n 24), p2.

33 Words of Cooke J, *R v Helen**, 2012, (*supra* n 8) at para 2.

34 Words of Pepperall J, *R v Rosie**, 2023, (*supra* n 3) at para 24.

35 Words of Rafferty J, *R v Helen**, 2013, (*supra* n 4) at para 21.

36 Words taken from a letter written by Rosie’s* former partner, *R v Rosie**, 2023, (*supra* n 1), at para 35.

37 Words of Sharp, *R v Rosie**, 2023, (*supra* n 24), p. 2.

38 Hannah’s words*, quoted in BBC. 2025. ‘Illegal abortion accused ‘shocked’ by pregnancy’. *BBC News Online.* 1 May. [Accessed 19.05.25].

39 For example, see British Medical Association. 2017.

40 Words of Rafferty J. *R v Helen**, 2013, (*supra* n 4), at paras 3 & 17 respectfully.

41 Words of Dan Cordey, the lawyer defending Lois*, in Hartley-Parkinson 2015.

42 Words of Sharp, *R v Rosie**, 2023, (*supra* n 1), at para 14.

43
*R v Helen**. 2013, (*supra* n 4), at para 16.

44 Words of Lady Justice Rafferty. *R v Helen**. 2013, (*supra* n 4), at para 16.

45 Words of Dan Cordey, the lawyer defending Lois*, in Hartley-Parkinson 2015.

46 Lois*, quoted in Hartley-Parkinson 2015.

47 Words taken from a letter written by Rosie’s* former partner, read out in court as part of her appeal case, *R v Rosie**, 18 October 2023.

48 Words taken from a letter written by Rosie’s* former partner, *R v Rosie**, 2023, (*supra* n 1), at para 35.

49
*R v Rosie**, 2023, (*supra* n 1), at para 14.

50 Words of Sharp, *R v Rosie**, 2023, (*supra* n 24), p.3.

51 Words of Sharpe. *R v Rosie**, 2023, (*supra* n 1), at para 50.

52 Mr Jolyon Perks, for the prosecution, quoted in Brown 2024.

53 CPS spokesperson, quoted in Al-Othman, Hannah. and Morris 2024.

54 Words of Judge Ian Lawrie KC, quoted in Oppenheim 2024.

55 Words of Recorder John Hardy KC reacting to the case of a woman who was charged more than a year after her baby was delivered by a C-section. During an examination by a doctor, two hexagonal tablets were found in the mother’s body, believed to be misoprostol, and so she was reported to the police. The baby survived and lived with the mother. The case was dropped on evidential grounds, and due to a lack of public interest. Recorder Hardy described the case as a ‘sad and tragic one’, labelling the prosecution ‘misconceived’ and the five-day trial a ‘waste of time’. Quote taken from Seaward 2022.

56 Words of Stella Creasy MP, quoted in Al-Othman and Morris 2024.

57 CPS spokesperson, quoted in Al-Othman, H and Morris, S, 2024.

58 Shropshire* police spokesperson, quoted in Davis 2024.

59 Tom Godfrey, defending Nancy* in court, describes how she has been ‘retraumatised’ by the court process. A letter from Nancy’s* friend, read out in court, described her as ‘completely broken’. Quoted in Al-Othman, H and Morris, S, 18 December 2024, *The Guardian.*

60 CPS spokesperson, quoted in Al-Othman and Morris 2024.

61 Nicholas Lumley KC, the barrister representing Chloe*, describing his client, quoted in Brown 2024.

62 Words of Sammy*, quoted in BBC File on 4. 2024.

63 Sheldon, Sally. and Lord, Jonathan. 2023.

64 Alexandra Felix KC in the case of Hannah*, quoted in Boyd 2025.

65 Words of Hannah*, quoted in Al-Othman 2025.
